# Design of a SMA-based soft composite structure for wearable rehabilitation gloves

**DOI:** 10.3389/fnbot.2023.1047493

**Published:** 2023-02-10

**Authors:** Qiaolian Xie, Qiaoling Meng, Wenwei Yu, Zhiyu Wu, Rongna Xu, Qingxin Zeng, Zhongchao Zhou, Tianyi Yang, Hongliu Yu

**Affiliations:** ^1^Institute of Rehabilitation Engineering and Technology, University of Shanghai for Science and Technology, Shanghai, China; ^2^Shanghai Engineering Research Center of Assistive Devices, Shanghai, China; ^3^Department of Medical Engineering, Chiba University, Chiba, Japan; ^4^Center for Frontier Medical Engineering, Chiba University, Chiba, Japan

**Keywords:** soft exoskeleton, shape memory alloys (SMA), soft composite structure, hand exoskeleton, SMA actuator

## Abstract

The combination of smart soft composite structure based shape memory alloy (SMA) and exoskeleton technology has the advantages of light weight, energy saving, and great human-exoskeleton interaction. However, there are no relevant studies on the application of SMA-based soft composite structure (SSCS) in hand exoskeletons. The main difficulty is that directional mechanical properties of SSCS need to comply with fingers movement, and SSCS can deliver enough output torque and displacement to the relevant joints. This paper aims to study the application of SSCS for wearable rehabilitation gloves and explore its bionic driving mechanism. This paper proposes a soft wearable glove (Glove-SSCS) for hand rehabilitation actuated by the SSCS, based on finger force analysis under different drive modes. The Glove-SSCS can support five-finger flexion and extension, weighs only 120 g, and adopts modular design. Each drive module adopts a soft composite structure. And the structure integrates actuation, sensing and execution, including an active layer (SMA spring), a passive layer (manganese steel sheet), a sensing layer (bending sensor) and connection layers. To obtain a high-performance SMA actuators, the performance of SMA materials was tested in terms of temperature and voltage, temperature at the shortest length, pre-tensile length and load. And the human-exoskeleton coupling model of Glove-SSCS is established and analyzed from force and motion. The results show that the Glove-SSCS can realize bidirectional movements of fingers flexion and extension, with ranges of motion are 90–110° and 30–40°, and their cycles are 13–19 s and 11–13 s. During the use of Glove-SSCS, the temperature of gloves is from 25 to 67°C, and the surface temperature of hands is from 32 to 36°C. The temperature of Glove-SSCS can be kept at the lowest temperature of SMA operation without much impact on the human body.

## 1. Introduction

**FIGURE 1:**
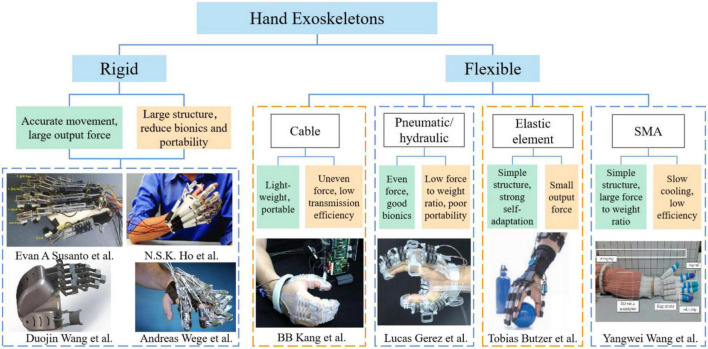
The state of hand exoskeletons.

Hand dysfunction is the most common and difficult functional disorder in the sequela of stroke. In the past decade, more and more rehabilitation training robots have been applied in the rehabilitation of hand dysfunction, and research on robotic training has proved that patients can enhance the hand motor function by performing repetitive rehabilitation training assisted by robots (Popov et al., 2016; Yao et al., 2017; Li et al., 2020). In terms of structure, hand exoskeletons can be divided into rigid hand exoskeletons and flexible (soft) hand exoskeletons. The rigid hand exoskeleton is mostly a connecting rod transmission mechanism. Although it has good robustness and motion accuracy, its rigid structure cannot adapt to the size and physiological state of different hands, and its complex mechanical structure and stiffness characteristics also reduce the human-machine compatibility, wearing comfort and portability of the device (Wege and Hommel, 2005; Ho et al., 2011; Susanto et al., 2015; Wang et al., 2018). In Figure 1, the shapes of soft hand exoskeletons are similar to gloves, and they have become the research focus of rehabilitation robotics with the advantages of simple structure, flexibility and lightness, simple assembly and low cost. The characteristics of hand exoskeletons are shown in Table 1.

**TABLE 1:** Characteristics of hand exoskeletons.

Developer	Force transmission	Weight	Fingers	Joints/DOF	Thumb	Compliance to finger joint	ROM	Sensor
N.S.K. Ho et al.	Linkages and linear actuator	Exoskeleton 500 g	4	MCP and PIP/ 2	No	Yes, finger joints moved along the circular slide	MCP-55°, PIP-65°	EMG
Andreas Wege et al.	Linkages, cables and DC motor	n/a	4	MCP, DIP and PIP/ 4	No	No	Ab/Ad: MCP −20∼20°, Fl/Ex: MCP −10∼90°, PIP 0∼90°, DIP 0∼45°	Hall sensors, quadrature encoders, motor currents, and force sensors
Duojin Wang et al.	Linkages and motor	n/a	5	MCP and PIP/ 2	Yes	Yes, rigid linkage structure coincides with rotation axis	MCP-65°, PIP-65°	EMG
Evan A Susanto et al.	Linkages and linear actuator	Exoskeleton 489 g	5	MCP and PIP/ 2	Yes	Yes, finger joints moved along the circular slide	MCP-55°, PIP-65°	EMG, 9 full-bridge strain-gauges,and sliding potentiometers
B B Kang et al.	Cable and motor	Exo-Glove Poly I: actuator 1.63 kg; Exo-Glove Poly II glove 104 g and actuation 1.14 kg	3	MCP, PIP, DIP/ 3	Yes	Yes	n/a	EMG, a bending sensor, and a mat-type pressure sensor
Sung-Sik Yun et al.	Actuator modules and three air pumps	Total 600 g (350 g without its case)	3	MCP, PIP, DIP/ 3	Yes	Yes	n/a	n/a
Tobias Butzer et al.	Two DC motors, cable and three layered sliding spring mechanism	Glove 148 g/ Total 640 g	5	MCP, PIP, DIP, CMC/ 4	Yes	Yes	Fl/Ex: finger −15°∼ 90°, thumb −10°∼ 45°, Ab/Ad: finger 0∼20°	Bending angle sensor
Yangwei Wang et al.	SMA wire and pulley mechanism	n/a	5	MCP, PIP, DIP/ 3	Yes	Yes	MCP 45°, PIP 62°, DIP 71°	Pressure sensor

DOF, degree of freedom; ROM, range of motion; MCP, metacarpophalangeal joint; PIP, interphalangeal joint; DIP, distal interphalangeal joints; CMC, carpometacarpal joint; EMG, electromyograph; Ab, abduction; Ad, adduction; Fl, flexion; Ex, extension.

The flexible (soft) hand exoskeleton has four main structural types in Figure 1: cable-driven mechanism, pneumatic/hydraulic drive, elastic element drive, and SMA drive. Flexion and extension training is essential for hand rehabilitation. Finger extension is particularly vital for stroke patients who have spastic flexion of the hemiplegic hand. Cable drive is a common way method of actuation for flexible hand exoskeletons. For example, a research team from Seoul National University designed a bidirectional cable-driven assistive glove (In et al., 2015; Kang et al., 2016), Mohammadi et al. (2018) from University of Melbourne developed a wearable exoskeleton driven by motor and cable, and a cable-driven flexible hand exoskeleton designed by Xiloyannis et al. (2016). However, the cable drive is generally used to drive the finger flexion only in one direction, and the finger extension mostly adopts other driving mechanisms. In addition, the cable-driven hand exoskeletons have some problems, such as low transmission efficiency and unwanted residual forces, which may cause some discomfort to the wearer. The pneumatic/hydraulic soft composite structure has the characteristics of flexibility and large output torque, but its weight is increased due to the additional force source required for pneumatic/hydraulic operation. Such as the pneumatic soft composite hand exoskeleton developed by Gerez et al. (2020), the hydraulically driven flexible hand exoskeleton designed by Polygerinos et al. (2015) of Harvard University, the fabric-based bidirectional pneumatic flexible hand exoskeleton developed by Yap et al. (2016, 2017), National University of Singapore, and the pneumatic flexible hand exoskeleton proposed by Yun et al. (2017), Seoul National University. Flexible hand exoskeletons driven by elastic elements can be lightweight and highly adaptive, but their output torque is small due to the limitation of material properties. For example, the pusher type flexible hand exoskeleton designed by Bützer et al. (2021), ETH Zurich, and the flexible hand exoskeleton named Mano developed by Randazzo et al. (2017).

Wang et al. (2021) introduced a flexible hand movement device driven by shape memory alloy (SMA) wires, which is compact, lightweight, and easy to use in activities of daily living (ADL). Park et al. (2020) presented a novel fabric muscle based on SMA springs, which generated a maximum contraction strain of 67%. Jeong et al. (2019, 2021) proposed a flexible wrist exoskeleton actuates by SMA springs, the exoskeleton has high shrinkage strain capacity. The wearable mass of the exoskeleton is 151 g, and the maximum torque is 1.32 N.m. SMA materials are directly driven as actuation components in these SMA smart structures, which can generate linear motions. However, this actuation method may cause some problems in the wearable robots, such as stress concentration and poor comfort, similar to cable drive and linear motor drive. The other is to embed SMA materials into composite structures.

Shape memory alloys embedded composite structure, which is known as smart soft composite structure has been studied to achieve a larger bending displacement and complex motions (Lee et al., 2020). SMA-based soft composite structure (SSCS) has been favored by many scholars in the research of bionic robots because of its good mechanical performance, programmable drive and multiple driving dimensions (Kim et al., 2012; Rodrigue et al., 2017; Lee et al., 2019). In addition to the above characteristics, the robot based on SSCS also has a high force-to-weight ratio, which indicates that it has a great application prospect in exoskeleton robots. At present, SSCS applied in the rehabilitation robots mostly focuses on the study of elastic actuation [e.g., artificial muscle (Zhang, 2014)]. However, there are no relevant studies on the application of SSCS in hand exoskeletons. The main difficulty is that directional mechanical properties of SSCS need to comply with fingers motions, and SSCS can provide enough output torque and displacement to the relevant joints. Therefore, it is essential to explore the bionic actuation mechanism through the mechanical characteristics and human-exoskeleton model of SSCS.

This paper aims to explore the application of SSCS in hand exoskeleton rehabilitation robots and its bionic actuation mechanism. This paper proposes a wearable glove for hand rehabilitation actuated by SSCS (Glove-SSCS), based on finger force analysis under different drive modes. To obtain high performance SMA actuators, the performance of SMA materials was tested in terms of temperature and voltage, the temperature at which the SMA contracts to the shortest length (TSL), pre-tensile length and load. The human-exoskeleton coupling model of the hand exoskeleton is established to analyze the human-exoskeleton force and motion angle. Lastly, the driving performance of SSCS is verified by finger motion angle and temperature experiments.

## 2. Design of a soft and wearable exoskeleton for hand rehabilitation actuated by SMA-based soft composite structure (Glove-SSCS)

Based on the current research status of hand exoskeletons and the movement characteristics of human hands, the design requirements of soft wearable exoskeleton for hand rehabilitation are summarized:

1. Light weight and portability, easy to wear, improve the wearing efficiency and enhance the wearing comfort of exoskeleton.
2. Optimize parameters of the individual differences of the human body, which can optimize the parameters of the exoskeleton according to the needs of different patients and different joints.
3. Bionic motion and stretching performance, good stretching performance can enable patients with hand spasticity to achieve stretching exercise and relieve spasticity.
4. Human-exoskeleton interaction, the exoskeleton and hand can keep coordinated movement, and reduce uncomfortable gestures.

### 2.1. Finger force analysis under different drive modes

**FIGURE 2:**
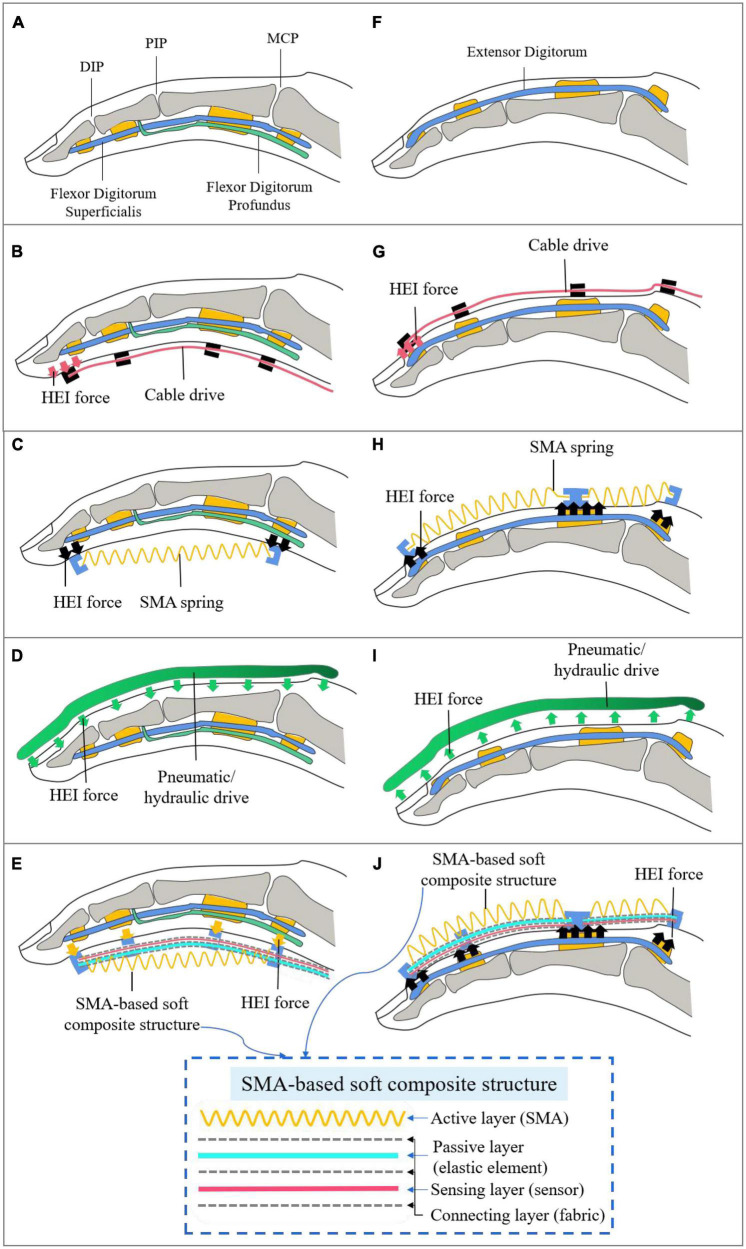
Finger force models in different drive modes. HEI, human-exoskeleton interaction. **(A,F)** The model of the bones and muscles of finger flexion and extension; **(B,G)** the cable-actuated force model of finger flexion and extension; **(C,H)** the force model of the SMA spring contraction directly actuating the finger movement; **(D,I)** the force model driven by pneumatic actuators of finger flexion and extension; **(E,J)** the force model of the driving finger driven by SMA-based soft composite structure.

All fingers except the thumb have three joints and four bones. The joints include metacarpophalangeal (MCP), proximal interphalangeal (PIP), and distal interphalangeal (DIP) joints. There is a strong coupling between PIP and DIP, that is, there is a relationship between the movement angles of PIP and DIP. The bones consist of the metacarpal, proximal phalange, intermediate phalange, and distal phalange. The thumb has only two joints and two phalanges. Figure 2A is a model of the bones and muscles of finger flexion. The active muscle groups for finger flexion refer to the flexor digitorum profundus and flexor digitorum superficialis.

The force model of the cable directly driving the finger flexion (without being incorporated elastic structures) is shown in Figure 2B. The cable drive simulates the movement of flexor muscles. The cable is arranged on the skin of the finger, the anchor point is set at the fingertip, and other fixed points are set parallel to the phalanges. The cable drive transmits tension by shortening the length of the cable, and the tension is transferred to the fingertips, creating joints motion from far to near, and ultimately bending the fingers. The cable drive transfers the fingertip force back, which creates a concentration of stress in the fingertip. It may cause some discomfort to the wearer, such as unwanted residual forces (Vidal et al., 2021). And the finger is straight and parallel to the direction of the cable tension, the cable drive is not easy to pull the finger movement.

The force model of finger flexion driven by pneumatic actuators, as shown in Figure 2D, requires the design of a cavity that meets the needs of finger movement. Hydraulic actuators are similar to pneumatic actuators. Different from the cable drive, pneumatic exoskeletons mostly place air cavities on the back of the hand, which has the advantage of not limiting movement space on the inside of the fingers. When the air cavity is ventilated, pressure is generated on the contact surface with the finger to drive the finger to flex, which avoids the problem of stress concentration caused by cable drive. Pneumatic exoskeletons also have a large output torque, strong compliance and other advantages. However, it is hard to achieve a high control accuracy due to the high non-linearity of the pneumatic drive process. Pneumatic actuators require additional power, which reduces the portability of the soft exoskeleton and makes it hard to simultaneously perform light and multiple degrees of freedom (DOF) hand rehabilitation training.

To realize the driving requirements of the soft hand exoskeleton with multiple DOF and lightweight portability, a new type of intelligent material SMA is adopted in this paper to replace the traditional actuation method of soft hand exoskeletons. The temperature-controlled SMA material can generate contractive force after heating. The material has the advantages of good flexibility, simple driving, lightweight portability, rapid response and high-power ratio, and is a potential bionic muscle material. The common shapes of SMA materials are wire, spring and sheet, etc. The SMA wire can generate a large force, but the strain of SMA wire is relatively low (<8%), which is difficult to meet the movement requirements of soft exoskeletons. The SMA spring can generate more than 200% strain (Xie et al., 2021), and it becomes a potential material for soft exoskeletons due to the large deformation characteristic. The force model of the SMA spring contraction directly actuating the finger movement is shown in Figure 2C. The contraction of SMA spring produces tension on the finger at both ends and drives the finger to flex. The way that the SMA spring actuating the finger is similar to the cable drive. To avoid the problem of stress concentration caused by cable drive, this paper proposes a soft composite structure, including an active layer (SMA), a passive layer (elastic element), a sensing layer (sensor), and connection layers (fabric). The force model of the driving finger driven by the composite structure is shown in Figure 2E. The composite structure can transform the axial contraction motion of SMA into the bending motion of the elastic element. Bending motion can make the finger force uniform, in contact with the fingers can generate tension on the surface. The way that the SMA composite structure drives the finger is similar to the pneumatic actuator, which can make the finger force more uniform. Compared with the pneumatic extra power, the SMA uses a simpler electric heating drive that enhances the portability of the soft hand exoskeleton.

Compared to actuating the fingers to flex, it is more difficult to actuate the fingers to extend. The primary muscles for finger extension refer to extensor digitorum (Figure 2F). If the cable drive mimics the finger extension muscles to place the cable on the back of hands, it is impossible to pull the finger to perform the extension because the stress concentration of the fingertip will cause the DIP joint to be extended and the PIP joint to be flexed (Figure 2G). The cable-driven soft hand exoskeletons can use elastic elements to store energy to perform finger extension movements. The cable is placed in the palm and the elastic element is placed on the back of hands. When the cable pulls the finger to flex, the elastic element deforms and stores energy. When the cable stops pulling, the elastic element releases energy to complete the finger extension. The way that the SMA spring actuating the finger is similar to the cable drive (Figure 2H). The pneumatic actuator with bidirectional motion can make the finger flexion and extension (Figure 2I). Finger extension requires more force than flexion, so two SMA springs are used to actuate the finger extension movement, one SMA to actuate the DIP and PIP joints, and one SMA to actuate the MCP joint. This is designed to increase power output and prevent the SMA from overstretching. Similar to the design of SMA-actuated flexion, the extended actuator is a SSCS (Figure 2J). The SMA spring contracts to actuate the elastic element and produces an upward force to drive the finger. Furthermore, the SMA spring is elastic, so the stretching of the hand spasms does not generate a significant force and can avoid secondary injury to the patient.

To sum up, the SSCS in Figures 2E, J can solve stress concentration fingertip of cable drive and poor portability of pneumatic actuator, and achieve the flexibility, light weight, portability, simple drive, high power ratio, and multiple DOF.

### 2.2. Design of a soft hand exoskeleton actuated by SMA-based soft composite structure

**FIGURE 3:**
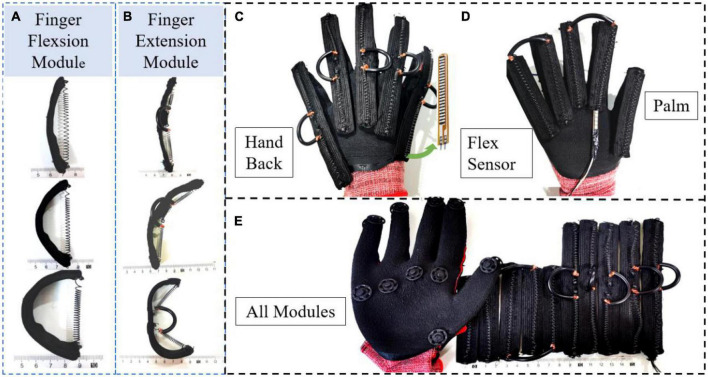
Design of a soft hand exoskeleton actuated by SMA-based soft composite structure. **(A,B)** Finger flexion and extension modules; **(C,D)** the design of the back and palm of the soft hand exoskeleton; **(E)** the modular design of the soft hand exoskeleton.

**FIGURE 4:**
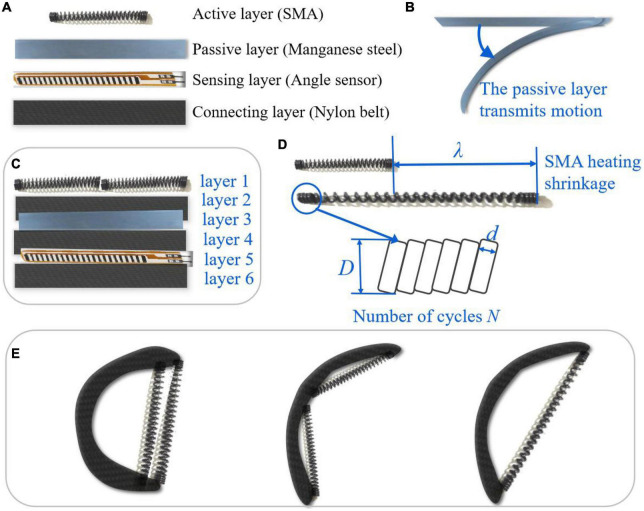
SMA-based soft composite structure. **(A,C)** The design of SMA-based soft composite structure (SSCS); **(B)** the passive layer; **(D)** parameters of SMA springs; **(E)** different configurations of SSCS.

The soft hand exoskeleton designed based on the SSCS in Figures 2E, J is shown in Figure 3. The hand exoskeleton has a modular design of five finger flexion modules (Figure 3A) and five finger extension modules (Figure 3B), each of which is fastened to the glove by hidden buckles (Figure 3C). Figures 3D, E show the design and operating principle of finger extension and flexion of the soft hand exoskeleton. The SMA spring is the active element that drives finger movement. The active driving force can be generated by heating SMA springs, and the dashed arrows in Figures 3D, E show the direction of SMA spring contraction. The manganese steel sheet combined with the SMA spring can bend and deform to transmit motion to the finger, causing the finger to flex and extend. The hand exoskeleton is lightweight and weighs only 120 g. Each actuator module adopts SSCS, which is a new type of flexible bionic joint integrating actuation, sensing, and execution. The bionic joint structure of SSCS is designed by combining the finger motion characteristics, including active layer, passive layer, sensing layer, and connecting layers, as shown in Figure 4. The active layer is the SMA spring, and its function is to provide driving force. The passive layer is the manganese steel sheet, and its function is to transmit motion, that is, to convert the axial contraction motion of the SMA spring into bending motion. The sensing layer is a bending sensor and its function is to measure the bending angle of the composite structure, and this layer is not mandatory for SSCS and is determined by design requirements. In addition, the resistance value of SMA springs changes with phase change and therefore their resistance value can also be used as sensing for SMA springs, but it necessary to establish the model of resistance and bending angle of SSCS. The connecting layers are the nylon belt, and its function is to connect the active layer, passive layer and sensing layer. The directional mechanical properties of SSCS can conform to the movement of finger joints, and can transfer torque and displacement to the corresponding joints. The active layer of flexion module has one SMA spring, while the active layer of extension module has two SMA springs. This is because the extension module requires a longer SMA length than the flexion module during the finger flexion movement, which is generated by change of rotation centers of the finger joints. Whereas the SMA springs cannot be extended indefinitely, the SMA length required for the extension module is outside the length range of the SMA, which was experimented with as shown in Section “2.3.3. Pre-tensile length of SMA springs.” The flexion module is not outside the length range of SMA, so the extension module has two SMA springs and the flexion module has only one SMA spring.

The SMA spring is heated by electricity. The minimum voltage to actuate one SMA spring is about 2 V, and the value of voltage will affect the speed of the contraction of the SMA spring. Each actuator module can be controlled independently, which can improve the DOF of hand exoskeleton and realize different gestures. The working principle of the actuator module is realized by using the shape memory efficiency of SMA. After heating the SMA spring, its internal crystal structure changes due to the temperature rise. It is the martensite phase at low temperature, and it is the austenite phase at high temperature. At the same time, SMA outputs contraction force and displacement. In the SSCS, the SMA spring is heated to produce contraction motion and drive the deformation of the elastic element, while the SSCS produces bending motion and transfers the force to the glove to actuate the finger movement. After the SMA spring stops heating, the energy stored by the elastic element deforms and releases energy to help the finger return to its original position.

**FIGURE 5:**
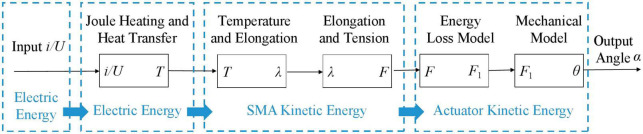
Energy transfer process of the SMA-based soft composite structure. i/U: current/voltage, T: temperature, λ: elongation of SMA spring, F: contraction force of SMA spring, F1: force of manganese steel, θ: angle of SSCS, α: angle of finger.

Many scholars have studied the mechanism of SMA. Combined with the SSCS, the energy transfer process of SMA is shown in Figure 5. The energy transfer process of SSCS: electric energy is converted into heat energy, and heat energy is converted into kinetic energy. One part of kinetic energy of the SMA spring is converted into the potential energy of the elastic element, which transmits motion through deformation and releases energy to make the SMA spring return to the initial state. The other part of the energy is converted into kinetic energy to drive the finger movement.

### 2.3. Performance test of shape memory alloy

**FIGURE 6:**
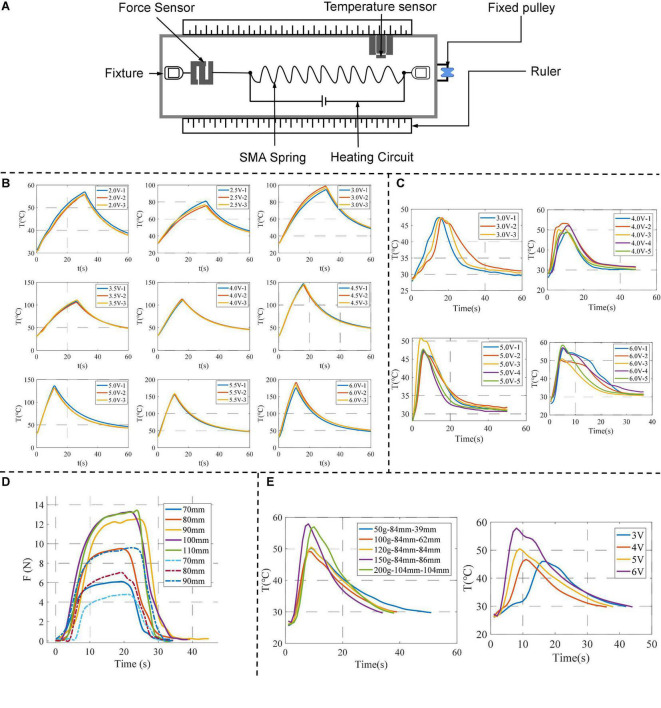
Performance test of SMA springs. **(A)** The performance test platform; **(B)** temperature variation of SMA springs driven by different voltages; **(C)** the results of repeated experiments of TSL under different voltages; **(D)** the experimental results for SMA springs with different pre-tensile lengths; **(E)** the results of experiments of load, the left figure shows the temperature change of different loads under the same voltage (5 V), and the right figure shows the temperature and time change of different voltages under the same load (120 g).

Currently, the biggest problem limiting the application of SMA actuators is the slow heating and cooling rate, which affects their working efficiency. Based on the energy transfer process of SSCS, this paper lists four key factors that affect the working efficiency of SMA, including temperature and voltage, TSL, pre-tensile length and load. The performance test platform is shown in Figure 6A, which consists of a tension sensor, a temperature sensor, a heating device, a pulley and weights. The actual effect of each factor on SMA springs is determined by experiments, which can provide a reference for improving the working efficiency of SMA actuators. The parameters of SMA springs used in the experiments are shown in Table 2.

**TABLE 2:** The parameters of SMA springs.

Symbol	Value	Symbol	Value
Initial length λ_0_	20 mm	Wire diameter *d*	0.5 mm
Coil diameter *D*	4.1 mm	Number of cycles *N*	22

#### 2.3.1. Temperature and voltage

For the SMA spring, temperature is an important parameter to be controlled, and the heating and cooling rate of the SMA determines the working efficiency of the SMA actuator. According to the first law of thermodynamics, the relationship between SMA temperature and current can be expressed as


(1) $$m\,c\,\frac{d\,T}{d\,t}=R\,I^{2}-h\,A\,\left(T-T_{0}\right)$$

Where *m* is the mass per unit length of SMA, *c* is the specific heat capacity, *R* is the resistance value, *I* is the current value, *h* is the thermal conductivity coefficient, *A* is the heat dissipation area per unit length of SMA, *T* is the temperature of SMA, and *T*_0_ is the room temperature. The room temperature for all experiments in this paper was about 23°C, and the larger the difference between the SMA temperature and room temperature, the faster the cooling rate of SMA.

The temperature test device of SMA is shown in Figure 6A. It was heated by electricity at different voltages, and then cooled by power off after heating for a few seconds. The experiment was repeated several times under the same experimental conditions, and the change curve of temperature *T* of SMA with time *t* was recorded.

The experimental results are shown in Figure 6A. The time corresponding to the maximum value of Y-axis temperature is the start of cooling. The results show that:

1. Under the same voltage, the temperature of SMA increases with time.
2. At the same heating time, the higher the voltage, the higher the temperature.

Therefore, increasing the driving voltage of SMA can increase the heating speed and enhance the working efficiency of SMA actuator. The driving voltage range of a single SMA spring is 4–6 V.

#### 2.3.2. Temperature at which the SMA contracts to the shortest length (TSL)

When the SMA spring is heated to the temperature at which the phase transition begins, the SMA spring begins to contract. When the temperature reaches the temperature at which the phase transition ends, the SMA spring ends to contract. The shorter the time for the SMA spring to reach the phase transition temperature, the shorter the time required for the SMA actuator to generate maximum deformation, thus increasing the heating rate of the SMA actuator.

The TSL test setup for SMA spring is shown in Figure 6B. The SMA spring was heated by electricity at different voltages. When the SMA spring is observed to contracted to its minimum length, the power is cut off and the air cooling is started at the same time. The experiment was repeated several times under the same conditions, and the change curve of temperature *T* of the SMA spring with time *t* was recorded.

The experimental results are shown in Figure 6C. The maximum temperature is the value of TSL. The results show that:

1. With the increase of voltage, the time of TSL decreases, and the value of TSL slightly increases, ranging from 45 to 60°C.
2. With air cooling, the temperature drops rapidly from TSL to 35°C, and from 35 to 30°C very slowly.

The value of TSL of the SMA spring obtained by experiments can be applied to the temperature control of SMA actuators, that is, when the temperature reaches TSL, the SMA spring reaches the shortest length. And the cooling rate of SMA is slow only by air cooling alone, especially the process from 35 to 30°C which takes a long time.

#### 2.3.3. Pre-tensile length of SMA springs

For the SMA spring, the pre-tensile length determines the contraction force. The relationship between the pre-tensile length and the contraction force of the SMA spring (Park et al., 2020) is as follows:


(2) $$F=\frac{G\,d^{4}}{8\,D^{3}\,N}\,\lambda$$

Parameters of the SMA spring: *F* is the contraction force, *G* is the shear modulus, *d* is the wire diameter of the SMA, *D* is the coil diameter of the SMA, λ is the variation of the tensile length (the difference between the pre-tensile length and the initial length). According to equation (2), the larger the pre-tensile length, the greater the contraction force *F*. And the pre-tensile length of the SMA spring will also affect the heating time.

One end of the SMA spring was fixed to the test platform and the other end was connected to the tension sensor. The test platform is shown in Figure 6A. The SMA spring with an initial length of 20 mm is stretched to different lengths (70–110 mm) and heating it electrically. The tension sensor records the contraction force of the SMA spring.

The change curve of contraction force *F* under different pre-tensile lengths is shown by the solid line in Figure 6D. And the results show that:

1. With the increase of pre-tensile length, the time required to reach the maximum contraction force increases, and the maximum value of contraction force increases.
2. At a certain pre-tensile length, the contraction force of SMA changes from slow to fast to slow.

In this experiment, when the pre-tensile length of the SMA spring exceeds a certain value, the electric heating of the SMA spring after unloading cannot completely restore to the initial length, which is the maximum pre-tensile length. When the pre-tensile length is 110 mm and then the SMA spring is stretched again to 70, 80, and 90 mm, respectively, the change curve of the contraction force is shown by the dotted line in Figure 6D. The dotted lines are the contraction force curves after the loading and stretching of 110 mm, and the solid lines are the contraction forces before the loading and stretching of 110 mm. It is found that the maximum contraction force *F*_*max*_ of the same length decreases after over stretching.

Therefore, the optimal range of pre-tensile length of the SMA spring is 80–100 mm, and the corresponding contraction force is about 11–13 N. The pre-tensile length should be determined according to the actual placement position and contraction force of the SMA spring.

#### 2.3.4. Load

Indeed, load is one of the main factors that affect the working efficiency of SMA actuators. For different loads, the heating and cooling time of SMA springs are different. The purpose of the load experiment is to explore the relationship between heating and cooling time and load, and how much load the SMA can be designed to improve work efficiency. The load test setup of the SMA spring is shown in Figure 6A. The SMA spring is electrified and heated under different loads until it is shrunk to the shortest length, and then the power is cut off and cooled by air cooling. And switch to different voltages for experiments, record the SMA spring temperature *T* with time *t* change curve. The results of the load experiment are shown in Figure 6E, the left figure shows the temperature change of different loads under the same voltage (5 V), and the right figure shows the temperature and time change of different voltages under the same load (120 g). The time corresponding to the maximum temperature is the time to start cooling. According to the experimental results, it is found that:

1. The cooling time of the SMA spring is significantly shorter when the load is from 20 to 100 g, and the cooling time of the SMA spring is not changed significantly when the load is from 100 to 200 g.
2. The TSL time change of the SMA spring is not obvious under different loads. That is, the time change corresponding to the maximum temperature is not obvious, which is between 2 and 3 s.
3. The stretched length of the SMA spring after cooling increases as the load increases. When the load is 120 g, the SMA spring can approximately return to its pre-tensile length after cooling.

Therefore, the cooling rate of the SMA spring can be effectively accelerated when the load ≥ 120 g and can be approximately restored to the pre-tensile length after cooling.

### 2.4. Human-exoskeleton coupling model of hand exoskeleton actuated by SMA-based soft composite structure (SSCS)

#### 2.4.1. Energy storage of elastic components

**FIGURE 7:**
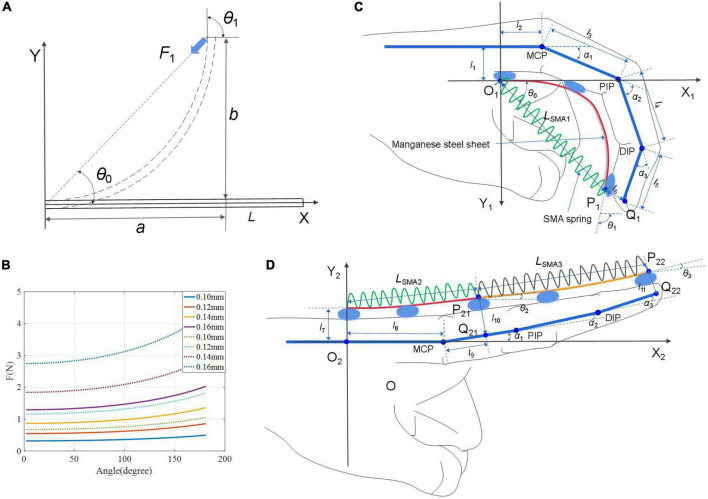
Human-exoskeleton coupling model. **(A)** Force model of manganese steel sheet; **(B)** the variation curve of *F*_1_ with the angle at different thicknesses; **(C,D)** the human-exoskeleton coupling model of finger flexion and extension.

In this paper, the manganese steel sheet can also be used as the elastic element for energy storage of SSCS. The heating and contraction process of the SMA spring is accompanied by the deformation and energy storage of the manganese steel sheet. When the SMA spring is cooled after power off, the manganese steel sheet releases the stored energy, so that the SMA spring can be restored to its original length and the cooling speed of the SMA spring can be accelerated at the same time. Therefore, the role of the manganese steel sheet is not only to transfer the motion but also to provide a restorative force to restore the SMA spring to its original length and accelerate the cooling rate. According to the load experiment, the SMA spring needs at least 120 g load, that is, about 1.2 N restoring force, so the design of deformation energy storage of manganese steel sheet should meet the restoring force required by the SMA spring. The model of force action on the free end of the manganese steel sheet is shown in Figure 7A, and the force and deformation can be written as (Howell, 2013):


(3) $$\theta_{1}=\frac{F_{1}\,L}{E\,I}\,\left[b\,\sin\theta_{0}+\left(L-a\right)\,\cos\theta_{0}\right]$$

The deformation in the vertical direction is


(4) $$\frac{F_{1}}{E\,I}\,\left[b\,\sin\theta_{0}+\left(L-a\right)\,\cos\theta_{0}\right]=\frac{d\,\theta }{d\,s}=\frac{d\,\theta }{d\,y}\,\frac{d\,y}{d\,s}$$

Due to $\frac{d\,y}{d\,s}=\sin\theta$, equation (4) can be simplified as


(5) $$\frac{F_{1}}{E\,I}\,\left[b\,\sin\theta_{0}+\left(L-a\right)\,\cos\theta_{0}\right]=\frac{d\,\theta }{d\,s}\,\sin\theta$$

And $I=\frac{h\,d^{3}}{12}$, equation (5) can be simplified as


(6) $$F_{1}=\frac{E\,h\,d^{3}\,\left(1-\cos\theta_{1}\right)\,\sqrt{2-1\,\cos\theta_{1}}}{12\,L\,b\,\left[b\,\sin\theta_{1}+\left(L-a\right)\,\left(1-\cos\theta_{1}\right)\right]}$$

*E* is Young’s modulus of the manganese steel. *I* is the moment of inertia of the cross section. *F*_1_ is the force of manganese steel sheet. *h* and *d* are the width and thickness of the manganese steel sheet. θ_*0*_ and θ_1_ are the bending angle describing the manganese steel sheet in Figure 7A. *L* is the length, *a* and *b* are the horizontal and vertical coordinates of the end point.

The parameters of the manganese steel sheet of the finger flexion module: considering the finger length, the pre-tensile length of the SMA spring was chosen to be 90 mm. According to the pre-tensile length experiment, when the pre-tensile is 90 mm, the contraction force *F* can be greater than 10.0 N, and part of the energy generated is stored in the manganese steel sheet as the restoring force, and the other part of the energy actuates the finger movement. According to the load experiment, the restoring force required by SMA spring is about 1.2 N, so the manganese steel sheet *F*_1_ > 1.2 N. As shown in Figure 7B, the full line represents the variation curve of *F*_1_ with the flexion angle at different thicknesses when the pre-tensile length *L* = 90 mm, and the thickness *d* ≥ 0.16 mm can meet the design requirements. The width of the five fingers is different (>10 mm), and the width of the manganese steel sheet *h* is chosen to be 7 mm. Therefore, the parameters of manganese steel sheet can be determined as *L* = 90 mm, *h* = 7 mm, *d* ≥ 0.16 mm.

The parameters of the manganese steel sheet of the finger extension module: each extension module contains two SMA springs, one SMA spring to actuate the DIP and PIP joints, and the other to actuate the MCP joint. The pre-tensile length of both SMA springs is 70 mm. According to the pre-tensile length experiment, when the pre-tensile length is 70 mm, the contraction force *F* of SMA springs is about 6.0 N. As shown in Figure 7B, the dotted line is the variation curve of *F*_1_ with the angle at different thicknesses when the length is 70 mm. The thickness *d* ≥ 0.12 mm can meet the design requirements. Therefore, the parameters of the manganese steel sheet of the extension module can be determined as *L* = 70 mm, *h* = 7 mm, *d* ≥ 0.12 mm.

#### 2.4.2. Human-exoskeleton coupling model

The human-exoskeleton coupling model of SSCS and finger is established, as shown in Figures 7C, D. Figure 7C is a model of finger flexion and Figure 7D is a model of finger extension. P_1_ and Q_1_ are the end points of the manganese steel sheet (or SMA spring 1) and the finger, respectively. The coordinate of P_1_ (x_*P1*_, y_*P1*_) is


(7) $$x_{\mathrm{P1}}=\frac{L_{1}\,\left(1-\cos\theta_{1}\right)}{\theta_{1}},y_{\mathrm{P1}}=\frac{L_{1}\,\sin\theta_{1}}{\theta_{1}}$$

Then *L*_*SMA1*_ can be written as


(8) $$L_{\mathrm{SMA1}}=\sqrt{x_{\mathrm{P1}}^{2}+y_{\mathrm{P1}}^{2}}=\frac{L_{1}\,\sqrt{2-2\,\cos\theta_{1}}}{\theta_{1}}$$

*L*_*i*_ and *L*_*SMAi*_ are the lengths of manganese steel sheets and SMA springs, *i* = 1,2,3. In (8), *L*_*SMA1*_ and θ_*1*_ are variables, so it is known that *L*_*SMA1*_ can calculate the bending angle θ_*1*_ of manganese steel sheet.

The coordinates of Q_1_ (x_*Q1*_, y_*Q1*_) can be written as


(9) $$\begin{array}{c} x_{\mathrm{Q1}}=l_{2}+l_{3}\,\cos\alpha_{1}+l_{4}\,\cos\left(\alpha_{1}+\alpha_{2}\right)+l_{5}\,\cos\left(\alpha_{1}+\alpha_{2}+\alpha_{3}\right)\\ y_{\mathrm{Q1}}=-l_{1}+l_{3}\,\sin\alpha_{1}+l_{4}\,\sin\left(\alpha_{1}+\alpha_{2}\right)+l_{5}\,\sin\left(\alpha_{1}+\alpha_{2}+\alpha_{3}\right) \end{array}$$

α_*1*_, α_*2*_, and α_*3*_ are the angles of motion of the MCP, DIP, and PIP joints, respectively. *l*_*j*_ is the length, j = 1–11. The relation between P_1_ (x_*P1*_,y_*P1*_) and Q_1_ (x_*Q1*_, y_*Q1*_) can be written as:


(10) $$\begin{array}{c} x_{\mathrm{P1}}=x_{\mathrm{Q1}}-l_{6}\,\cos\left(\pi -\left(\alpha_{1}+\alpha_{2}+\alpha_{3}\right)\right)\\ y_{\mathrm{P1}}=y_{\mathrm{Q1}}-l_{6}\,\sin\left(\pi -\left(\alpha_{1}+\alpha_{2}+\alpha_{3}\right)\right)\\ L_{\mathrm{SMA1}}=\sqrt{x_{\mathrm{P1}}^{2}+y_{\mathrm{P1}}^{2}} \end{array}$$

Substitute (9) into (10), and the variables of the equation include *L*_*SMA1*_, α_*2*_, andα_*3*_. There is a coupling relationship between the motions of α_*1*_, α_*2*_, and α_*3*_ and the ratio of motion angles is 1:2:1.5 (Kazeminasab et al., 2019). Therefore, given *L*_*SMA1*_, the bending angles α_*1*_, α_*2*_, and α_*3*_ of each finger joint can be calculated.

Similarly, we can get that


(11) $$\begin{array}{c} L_{\mathrm{SMA2}}=\frac{\sqrt{L_{2}^{2}\,{\left(1-\cos\theta_{2}\right)}^{2}+{\left(L_{2}\,\sin\theta_{2}\right)}^{2}}}{\theta_{2}}\\ L_{\mathrm{SMA2}}=\sqrt{{\left(l_{8}+l_{9}\,\cos\alpha_{1}-l_{10}\,\sin\alpha_{1}\right)}^{2}+{\left(l_{9}\,\sin\alpha_{1}+l_{10}\,\cos\alpha_{1}\right)}^{2}} \end{array}$$

(12) $$\begin{array}{c} L_{\mathrm{SMA3}}=\frac{\sqrt{L_{3}^{2}\,{\left(1-\cos\theta_{3}\right)}^{2}+{\left(L_{3}\,\sin\theta_{3}\right)}^{2}}}{\theta_{3}}\\ x_{\mathrm{Q22}}=l_{8}+l_{3}\,\cos\alpha_{1}+l_{4}\,\cos\left(\alpha_{1}+\alpha_{2}\right)+l_{5}\,\cos\left(\alpha_{1}+\alpha_{2}+\alpha_{3}\right)\\ y_{\mathrm{Q22}}=l_{3}\,\sin\alpha_{1}+l_{4}\,\sin\left(\alpha_{1}+\alpha_{2}\right)+l_{5}\,\sin\left(\alpha_{1}+\alpha_{2}+\alpha_{3}\right)\\ L_{\mathrm{SMA3}}=\sqrt{{\left(x_{\mathrm{Q22}}-l_{11}\,\sin\left(\alpha_{1}+\alpha_{2}+\alpha_{3}\right)\right)}^{2}\,\cdots \to }\\ \bar{+{\left(y_{\mathrm{Q22}}+l_{11}\,\cos\left(\alpha_{1}+\alpha_{2}+\alpha_{3}\right)\right)}^{2}}-L_{\mathrm{SMA2}} \end{array}$$

*θ*_*2*_ and *θ*_*3*_ are the bending angle of the manganese steel sheets in Figure 7D. Therefore, given *L*_*SMA*2_ and *L*_*SMA*3_, the angles *θ*_*2*_ and *θ*_*3*_, α_*1*_, α_*2*_, and α_*3*_ can be calculated.

## 3. Experimental results

### 3.1. Output force and displacement performance of SSCS

**FIGURE 8:**
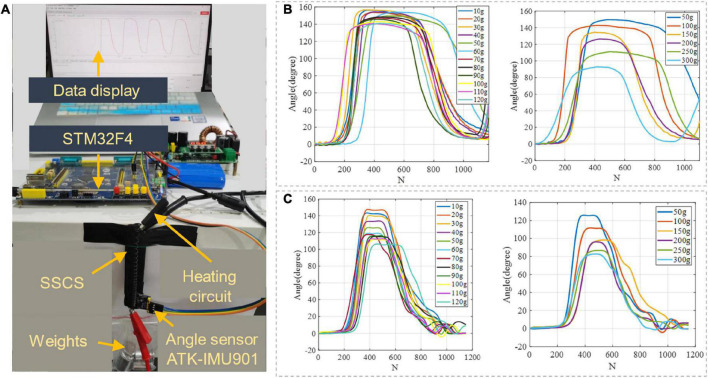
Output force and displacement performance of SSCS. **(A)** The experimental setup, **(B,C)** are the experimental results of SSCS with *d* = 0.1 and 0.2 mm, *N* (*N* = 62) is the number of samples.

To test the output force and bending displacement of SSCS with different thicknesses of manganese steel sheets, the experiment of output force and displacement performance was carried out in this paper. In this experiment, SSCS with two thicknesses of manganese steel sheets were selected to test their bending angles when driving objects of different weights. One end of the SSCS of the flexion module was fixed, and the other end was suspended with loads of different weights. Then, the SSCS was heated to complete contraction under 5 V voltage and 3 A current, and finally cooled to the initial state by power off. The angle sensor (ATK-IMU901) was used to record the motion angle of the SSCS, and the experimental setup is shown in Figure 8A. The experiment tested two sizes of SSCSs. The size of manganese steel sheet of SSCS1 was *L* = 90 mm, *h* = 7 mm, *d* = 0.1 mm. The size of manganese steel sheet of SSCS2 was *L* = 90 mm, *h* = 7 mm, *d* = 0.2 mm. The voltage and current of the experiment were 5 V and 3 A. The results of the SSCS1 and SSCS2 are shown in Figures 8B, C, respectively. When the loads are 10 to 120 g, the maximum motion angles of the SSCS1 do not change significantly from 140° to 160°, the maximum motion angles of the SSCS2 vary significantly from 106° to 150°. When the loads are 50 to 300 g, the maximum motion angles of the SSCS1 and SSCS2 change significantly from 90° to 150° and from 80° to 130°. Compared with the SSCS2, the motion range of the SSCS1 actuating the same loads is larger, because the thickness of manganese steel sheet of SSCS1 is thinner. And the motion angles of SSCS decrease with the increase of loads.

### 3.2. The current change of SSCS during heating

**FIGURE 9:**
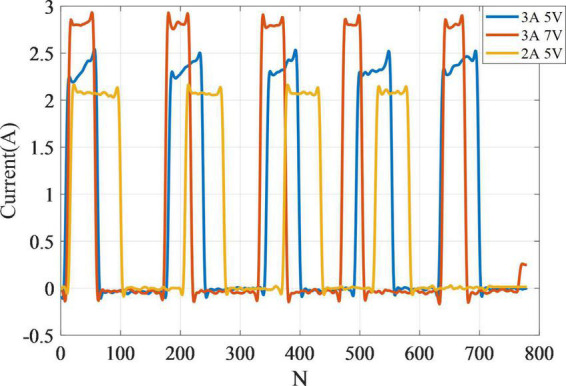
The current change of SSCS during heating.

To measure the resistance of the SSCS and its driving circuit, the experiment of current change of SSCS during heating was carried out in this paper. The SSCS of flexion module was heated from initial state to complete contraction under different voltages and currents, and the current detection module (ACS712) was used to record the current changes of the SSCS, as shown in Figure 9. The current of the SSCS increases from 2.2 to 2.5 A under voltage 5 V and current 3 A, which is due to the reduced resistance of SMA during heating. The resistance of the SSCS and its driving circuit can be calculated from this, and the resistance is 2.3 Ω in the initial state and 2 Ω when heated to complete contraction. The current of the SSCS does not change significant and remains 2.1 and 2.8 A under voltage 5 V and current 2 A, as well as voltage 7 V and current 3 A. The reason for this is that the current in the circuit reaches its limit, the current is unchanged and the voltage is changing. Therefore, this experiment can calculate the resistance of the circuit, and provide a reference for the selection of voltage and current of the SSCS driving circuit.

### 3.3. Temperature test and insulation of SSCS

**FIGURE 10:**
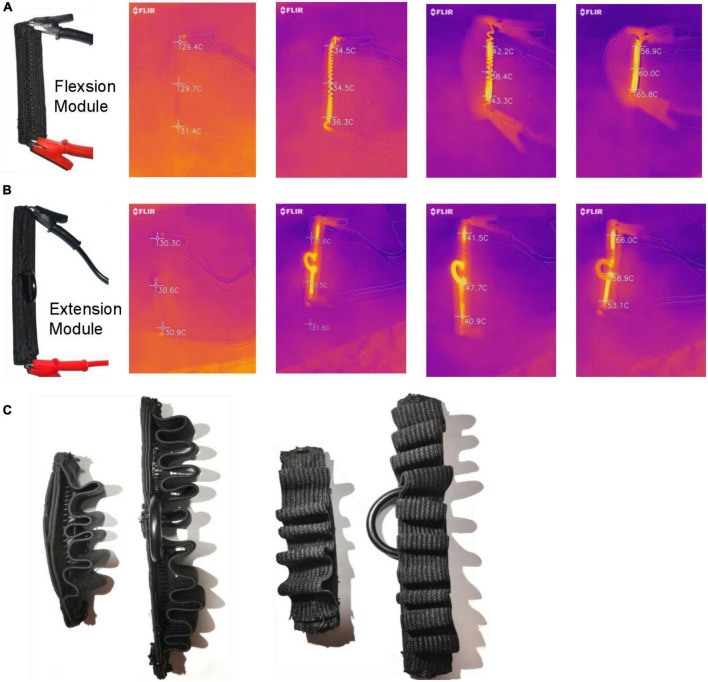
Temperature test and insulation of SSCS. **(A)** Flexion module, **(B)** extension module, **(C)** insulation.

The high temperature generated by SMA heating will be harmful to human skin. It is necessary to test the SMA temperature changes during SSCS operation and take the necessary insulation measures. The SSCS of flexion and extension modules was heated from initial state to complete contraction, and the FLIR ONE PRO infrared thermal imager was used to record the infrared thermal imaging. The results of flexion and extension modules are shown in Figures 10A, B. It can be seen from the results that the temperature of SMA varies from 29 to 66°C. A corrugated elastic band was used to cover the front of SSCS, namely, the direction of SMA contraction, as shown in Figure 10C, which could prevent the high temperature heated by SMA from scalding skin without hindering the movement of SSCS. In addition, the side of SSCS is still open, and can still be cooled by air cooling. This type of SSCS insulation avoids the risk of burning skin at high temperatures and can also be air-cooled.

### 3.4. Experiment of fingers motion angles

**FIGURE 11:**
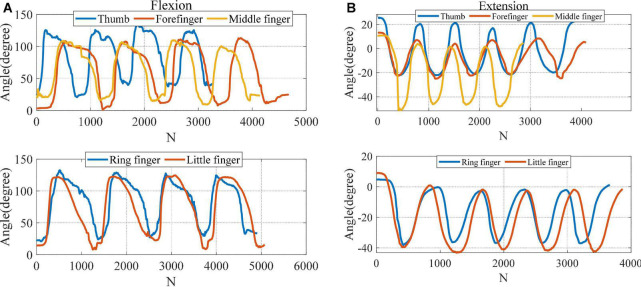
The motion angles of five fingers. **(A)** Flexion, **(B)** extension. *N* (*N* = 62) is the number of samples.

To verify the training effect of the exoskeleton glove, a healthy subject (female, 28 years old, 1.72 m in height, 67 kg in weight) was selected to test the motion angle and temperature changes. This study has been reviewed by Shanghai University of Medicine and Health Sciences ethics, batch number 2022-ZYXM4-04–420300197109053525. According to Section “3.1. Output force and displacement performance of SSCS,” the thickness of manganese steel sheet can affect the motion range of SSCS. The thickness of manganese steel sheet of flexion and extension modules is 0.1 mm to make the Glove-SSCS get more motion range. The subject wore the glove, and the angle sensor (ATK-IMU901) was fixed on the tip of every finger to record the data of fingers movement angles. During the flexion movement, the current was 3 A and the voltage was 5 V, the SMA was heated from the initial state to complete contraction, and then cooled to the initial state by power-off. The angles of five fingers flexion were recorded, as shown in Figure 11A. The motion range of thumb flexion is about 100° and its cycle is about 13 s. The motion range of forefinger flexion is about 105° and its cycle is about 17 s. The motion range of middle finger flexion is about 90° and its cycle is about 15 s. The motion range of flexion of the ring and little fingers is about 110° and its cycle is about 19 s. During the extension movement, the current was 3 A and the voltage was 7 V, the SMA was heated from the initial state to complete contraction, and then cooled to the initial state by power-off. The angles of five fingers extension were recorded, as shown in Figure 11B. The motion range of thumb extension is about 40° and its cycle is about 12 s. The motion range of forefinger extension is about 30° and its cycle is about 13 s. The motion range of middle finger extension is about 40° and its cycle is about 12 s. The motion range of extension of the ring and little fingers is about 40° and its cycle is about 12 s. To sum up, the Glove-SSCS can realize bidirectional movements of fingers flexion and extension, the motion ranges of fingers flexion and extension are 90–110° and 30–40°, and their cycles are 13–19 s and 11–13 s. It can be seen from the experiments that the flexion angle of Glove-SSCS is small, which is mainly caused by three reasons: (1) The inner glove worn is elastic, and the glove is deformed during the movement of the SSCS, so the glove consumes part of the deformation of the SSCS. (2) The role of the passive layer (manganese steel sheet) is to transmit the movement to the fingers, but it cannot fully comply with the movement of the finger joints, which limits the movement of the fingers. (3) The SMA spring of SSCS has fatigue effect. After repeated use, the SMA spring cannot shrink to its original length, and the motion angle of finger becomes smaller as the displacement deformation of the SMA spring becomes smaller.

### 3.5. Temperature measurement experiment of the exoskeleton glove and body surface

**FIGURE 12:**
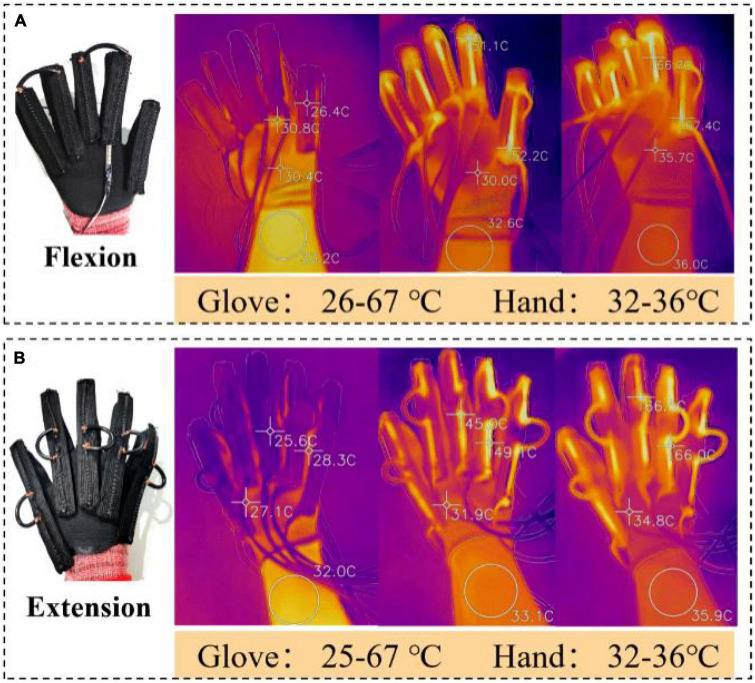
Temperature measurement experiment. **(A)** The maximum temperature of the SMA springs and wrist during hand flexion; **(B)** the maximum temperature of the SMA springs and wrist during hand extension.

The high temperature generated by SMA heating will be harmful to human skin. Therefore, it is necessary to judge the influence of SMA working process on the skin by temperature test. According to the phase transition mechanism of SMA, the threshold of phase transition temperature is about 65°C. A temperature sensor (GY-906-BCC) was used to monitor the real-time temperature of SMA springs. When the temperature of the SMA springs is lower than the threshold due to environmental factors, the voltage is increased to quickly restore to the contraction state. When the temperature reaches the threshold, the voltage is reduced to a smaller voltage to maintain the current temperature. As shown in Figure 12, the maximum temperature of the SMA springs is about 65°C during the flexion and extension of the exoskeleton, and the maximum temperature of the human wrist is about 36°C. In addition, the insulation method of SSCS in Section “3.3. Temperature test and insulation of SSCS” can further ensure the safety of users. As can be seen from Figure 6B, the temperature of SMA can exceed 150°C when heated by power for a long time, which will cause the risk of scalding and slow cooling due to excessive temperature.

### 3.6. Testing the fingertip force of the index finger and thumb of the exoskeleton glove

**FIGURE 13:**
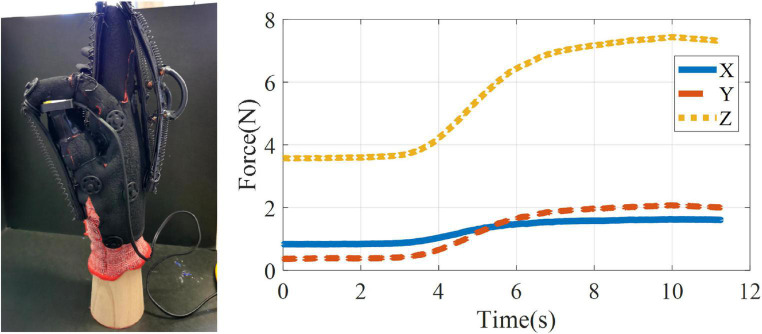
Testing the fingertip force of the index finger and thumb of the exoskeleton glove on a wooden hand.

The thumb and index finger are the two most commonly used fingers in daily life. In order to test the fingertip force of the index finger and thumb, a fingertip force test of the index finger and thumb of the exoskeleton glove was conducted on a wooden hand. The exoskeleton glove was placed on the wooden hand, and the force sensor (SC-2043-SG, Strain Gauge Measurement) was installed at the contact position of the index finger and thumb of the wooden hand, and the SSCS was heated (4 V) until the SMA was fully contracted, and the data of the force sensor for this process were recorded as shown in Figure 13. The force sensor has X, Y and Z axes, and the force signal is most obvious in the Z axis, which is vertical downward as a positive direction, with a maximum fingertip force of 7.4 N; the maximum fingertip forces in the X and Y axes are 1.7 and 2.0 N. The forces in the X and Y axes are related to the placement position of the force sensor.

## 4. Discussion

In the past decade, SMA materials have been successfully used in a variety of applications from biomedical fields to robotics and automation. The characteristics of SMA actuators, such as high power-to-weight ratio, simple structure, light weight and small size, make them an ideal alternative to pneumatic/hydraulic or motor drive. SMA actuators also have some shortcomings, such as slow cooling, low efficiency, insufficient strain or driving force, and time delay.

### 4.1. SMA actuator design

In this paper, a novel hand exoskeleton based on SMA soft composite structure is proposed. The soft composite structure includes an active layer (SMA spring), a passive layer (manganese steel sheet), a sensing layer (bending sensor), and connecting layers (nylon belt). The role of the active layer is to transfer force, the passive layer is used to transfer motion, the sensing layer is to measure the bending angle of the SSCS, and the connection layer is to connect each layer. The directional mechanical properties of SCSS can follow the movement of finger joints and transfer the output force and displacement to corresponding joints. Yao et al. (2017) proposed a lightweight hand exoskeleton that used SMA actuators and nylon strings to mimic the function of muscles and tendons. Wang et al. (2021) presented a hand exoskeleton actuated by SMA wire, and designed an extended-range mechanism to increase the strain of SMA wire. The above two SMA actuators are axial contraction motion, similar to cable or tendon drives. In this paper, the passive layer of SCSS can realize bending motion, and the motion of SCSS is more consistent with flexion and extension motion of finger joints than the axial contraction motion generated by direct SMA actuation.

### 4.2. Weight and modular design

Hand exoskeleton weight: Some researchers recommend that the maximum weight of the hand exoskeleton should not exceed 500 g (Araujo et al., 2021), and subjects with hand impairments suggested that a weight of 200 g on the hand was manageable (Bützer et al., 2021). The wearable part of the pneumatic hand exoskeleton proposed by Yap et al. (2017) weighs 150 g. Yao et al. (2017) presented a light weight soft muscle glove actuated by SMA spring weighing only 85 g. Bützer et al. (2021) presented sleek mechanisms for a fully wearable assistive soft hand exoskeleton, and the hand exoskeleton module only weighs 148 g. Popov et al. (2016) introduced a cable-driven portable exoskeleton glove with a weight of 250 g. The weight of the Glove-SSCS glove in this paper is only 120 g, which can also meet the weight requirements of the hand exoskeleton.

The Glove-SSCS is modular and has five SSCS driver modules in the palm and back of the hand. The advantages of modular design: (1) It can realize the control of different gestures by powering on and off different modules. (2) The personalized customization of patients is achieved by adjusting the design parameters of SSCS for patients with different conditions.

### 4.3. Fingers movement

Bidirectional training, namely, finger flexion, and extension, is crucial for hand rehabilitation, but at present, some flexible hand exoskeletons only provide finger flexion training, such as Radder et al. (2016) and Kim et al. (2017). In this paper, the SSCS are placed on the palm and the back of the hand to achieve the bidirectional movement of flexion and extension. However, placing the SSCS on palm will affect the grasp of objects. The soft composite structure actuated by pneumatic and hydraulic pressure is placed on the back of the hand, which provides a larger motion space for fingers. This exoskeleton has a strong hand-assisted grasp ability, which is suitable for assistance with activities of daily living of patients with hand dysfunction (Yun et al., 2017; Gerez et al., 2020). In contrast, the Glove-SSCS exoskeleton is more suitable for hand rehabilitation training.

To better train the motor function of hand, the movement angle of fingers needs to reach a certain range of motion (ROM). The flexion/extension range of finger joints in 90% of daily living activities is 19–71° MCP, 23–87° PIP, and 10–64° DIP, that is, the flexion/extension range of finger is 52–222° (Bain et al., 2014). In this paper, the motion ranges of fingers flexion and extension are 90–110° and 30–40°, and their cycles are 13–19 s and 11–13 s. The results show that the flexion angle of Glove-SSCS is small. In the follow-up work, the design of Glove-SSCS is improved from the following three aspects to increase the flexion angle: (1) The inner glove is replaced with a new material that is not easily deformed. (2) The design of the transfer layer should conform to the fingers movement. (3) Replace the failed SMA springs. In the follow-up work, the SSCS structure and size are optimized to increase its motion angle. In addition, the movement cycle of flexion and extension is about 3–5 times per minute, and a new cooling method should be developed to improve the efficiency of Glove-SSCS.

### 4.4. Security

The longer the heating time, the higher the temperature of SMA. Therefore, the temperature should be controlled at the lowest temperature for SMA operation. The maximum temperature of SMA springs can be controlled at about 65°C, and the highest temperature of human wrist is about 36°C, which can avoid the scald and discomfort caused by high temperature to the wearer and slow cooling. And the insulation method of SSCS in Section “3.3. Temperature test and insulation of SSCS” can further ensure the safety of users. In addition, the SMA spring also has the properties of springs, which is a feature that the motor drive does not have. For patients with excessive hand muscle tension, this feature of the SMA spring prevents the patients from causing injury (the maximum force of an SMA spring is about 14 N).

### 4.5. Control method

Polygerinos et al. (2015) presented a soft robotic glove actuated by hydraulic soft actuators. A sliding-mode controller was implemented, with a controller frequency of 20 Hz, the system was able to respond to the step input within 0.2 s (65%), and reach steady state after 2.2 s (95%). The closed loop system bandwidth (i.e., actuation speed of the glove actuators) was measured with a sinusoidal tracking performance test and found to be 0.25 Hz. Bützer et al. (2021) proposed a fully wearable assistive soft hand exoskeleton, it is actuated by compliant mechanisms, Bowden cables and DC motors. The two DC motors are controlled through force feed forward and actuate the flexion/extension of the fingers and the thumb, and the hand exoskeleton could open and close at a frequency of 0.5 Hz (one second to open and one second to close). The fingertips of thumb, middle finger, little finger are 5.4, 5.2, and 6.4 N, respectively. Villoslada et al. (2015) designed a SMA actuator that can be bent up to about 180° to provide more freedom of movements and a better integration in wearable robots, specially in soft wearable robots. The implemented PID control is based on a simultaneous velocity and position control for the SMA actuator, and the Hammerstein-Wiener model is used to model other non-linear systems aside from SMA actuators. The actuator follows the reference quite accurately at a velocity of 1,290 um/s, maintaining a constant position without oscillations. Jeong et al. (2021) proposed a soft wearable robot for assisting wrist motions with SMA muscles. The SMA muscle with mineral oil cooling system shows contraction time less than 1 s with 40% contraction ratio when 4 A current is applied for heating, and the proposed robot shows fast cyclic actuation performances of 0.1 Hz for flexion-extension motion and 0.13 Hz for radial-ulnar deviation motion. The general limitations of SMA actuators are non-linear operation, low actuation bandwidth, low efficiency, etc. In the follow-up work, the control study of Glove-SSCS will be carried out to improve the hand motion control.

### 4.6. Limitations

In summary, the Glove-SSCS in this paper still has some shortcomings. There are three main aspects:

1. The motion angle of SSCS is not enough, and the design of the flexion module will be improved in the follow-up to increase the flexion angle.
2. The driving frequency of SSCS can be further increased, and more efficient heating and cooling methods can be studied.
3. It is necessary to improve the control accuracy of Glove-SSCS to achieve precise hand motion control.

## 5. Conclusion

This paper proposes a hand exoskeleton for rehabilitation training of patients with hand dysfunction. We have developed a soft and wearable exoskeleton glove (Glove-SSCS), which uses SMA smart materials to replace traditional motors or pneumatic drives. The SMA spring can be driven by only energizing (2–6 V), which has the advantages of simple driving and large force-to-weight ratio. Meanwhile, the glove can support flexion and extension of five fingers, the wearable part only weighs 120 g, making it lightweight and portable. The following new elements were developed for the Glove-SSCS:

1. The application of a new smart soft composite structure in exoskeleton. This composite structure makes hand exoskeleton have the advantages of good bionics, simple drive and control, large force-weight ratio, lightweight and portability.
2. A soft composite structure integrating actuation, sensing and execution, including an active layer (SMA spring), a passive layer (manganese steel sheet), a sensing layer (bending sensor) and connection layers (nylon belt). The active layer is to transfer force, the passive layer is used to transfer motion, the sensing layer is to measure the bending angle of the SSCS, and the connection layer is to connect all layers.

This paper introduced several novel elements for a wearable rehabilitation glove. These elements provide solutions to certain problems of traditional wearable hand exoskeletons and can be used to develop other types of soft exoskeletons. There are still many challenges in the application of SSCS to exoskeletons, and it is necessary to continue to improve the mechanism innovative design and precise control of SSCS. For example, how to improve the SMA soft composite structure can meet the greater ROM of human joints, and how to further improve the SMA drive efficiency and control accuracy. In addition, the study results (angle and temperature) of one healthy subject were recorded in this paper, and the experiment should be conducted in a wider range of subjects and application scenarios.

## Data availability statement

The raw data supporting the conclusions of this article will be made available by the authors, without undue reservation.

## Ethics statement

This study has been reviewed by Shanghai University of Medicine and Health Sciences ethics, batch number 2022-ZYXM4-04–420300197109053525. The patients/participants provided their written informed consent to participate in this study.

## Author contributions

QX conceived the idea, developed the method, performed the experiments, and wrote the majority of the manuscript. QM, WY, and HY supervised and structured the manuscript. ZW and RX corrected the manuscript. QZ edited the manuscript. ZZ and TY performed the experiments. All authors contributed to the article and approved the submitted version.

## References

[B1] Araujo R. S. Silva C. R. Netto S. P. Morya E. Brasil F. L. (2021). Development of a low-cost EEG-controlled hand exoskeleton 3D printed on textiles. *Front. Neurosci.* 15:661569. 10.3389/fnins.2021.661569 34248478 PMC8267155

[B2] Bain G. I. Polites N. Higgs B. G. Heptinstall R. J. McGrath A. M. (2014). The functional range of motion of the finger joints. *J. Hand Surg.* 40 406–411. 10.1177/1753193414533754 24859993

[B3] Bützer T. Lambercy O. Arata J. Gassert R. (2021). Fully wearable actuated soft exoskeleton for grasping assistance in everyday activities. *Soft Robot.* 8 128–143. 10.1089/soro.2019.0135 32552422

[B4] Gerez L. Gao G. Dwivedi A. Liarokapis M. (2020). A hybrid, wearable exoskeleton glove equipped with variable stiffness joints, abduction capabilities, and a telescopic thumb. *IEEE Access* 8 173345–173358.

[B5] Ho N. Tong K. Hu X. Fung K. Wei X. Rong W. (2011). “An EMG-driven exoskeleton hand robotic training device on chronic stroke subjects: task training system for stroke rehabilitation,” in *Proceedings of the 2011 IEEE international conference on rehabilitation robotics*, Piscataway, NJ. 10.1109/ICORR.2011.5975340 22275545

[B6] Howell L. L. (2013). “Compliant mechanisms,” in *21st century kinematics*, ed. McCarthy J. (London: Springer).

[B7] In H. Kang B. B. Sin M. Cho K.-J. (2015). Exo-glove: A wearable robot for the hand with a soft tendon routing system. *IEEE Robot. Autom. Magaz.* 22 97–105. 10.1089/soro.2018.0006 30566026

[B8] Jeong J. Hyeon K. Han J. Park C. H. Ahn S.-Y. Bok S.-K. (2021). Wrist assisting soft wearable robot with stretchable coolant vessel integrated SMA muscle. *IEEE/ASME Trans. Mechatron.* 27 1046–1058.

[B9] Jeong J. Yasir I. B. Han J. Park C. H. Bok S.-K. Kyung K.-U. (2019). Design of shape memory alloy-based soft wearable robot for assisting wrist motion. *Appl. Sci.* 9:4025.

[B10] Kang B. B. Lee H. In H. Jeong U. Chung J. Cho K.-J. (2016). “Development of a polymer-based tendon-driven wearable robotic hand,” in *Proceedings of the 2016 IEEE International Conference on Robotics and Automation (ICRA)*, Piscataway, NJ, 3750–3755. 10.1089/soro.2018.0006

[B11] Kazeminasab S. Hadi A. Alipour K. Elahinia M. (2019). Force and motion control of a tendon-driven hand exoskeleton actuated by shape memory alloys. *Ind. Robot. Int. J.* 45 623–633.

[B12] Kim B. In H. Lee D.-Y. Cho K.-J. (2017). Development and assessment of a hand assist device: GRIPIT. *J. Neuroeng Rehabil.* 14 1–14. 10.1186/s12984-017-0223-4 28222759 PMC5319138

[B13] Kim H.-J. Song S.-H. Ahn S.-H. (2012). A turtle-like swimming robot using a smart soft composite (SSC) structure. *Smart Mater. Struct.* 22:014007.

[B14] Lee H. T. Seichepine F. Yang G. Z. (2020). Microtentacle actuators based on shape memory alloy smart soft composite. *Adv. Funct. Mater.* 30:2002510.

[B15] Lee J.-H. Chung Y. S. Rodrigue H. (2019). Long shape memory alloy tendon-based soft robotic actuators and implementation as a soft gripper. *Sci. Rep.* 9:11251. 10.1038/s41598-019-47794-1 31375746 PMC6677814

[B16] Li H. Cheng L. Li Z. Xue W. (2020). Active disturbance rejection control for a fluid-driven hand rehabilitation device. *IEEE/ASME Trans. Mechatron.* 26 841–853.

[B17] Mohammadi A. Lavranos J. Choong P. Oetomo D. (2018). “Flexo-glove: a 3D printed soft exoskeleton robotic glove for impaired hand rehabilitation and assistance,” in *Proceedings of the 2018 40th Annual International Conference of the IEEE Engineering in Medicine and Biology Society (EMBC)*, Piscataway, NJ. 10.1109/EMBC.2018.8512617 30440822

[B18] Park S. J. Kim U. Park C. H. (2020). A novel fabric muscle based on shape memory alloy springs. *Soft Robot.* 7 321–331. 31724903 10.1089/soro.2018.0107

[B19] Polygerinos P. Wang Z. Galloway K. C. Wood R. J. Walsh C. J. (2015). Soft robotic glove for combined assistance and at-home rehabilitation. *Robot. Autonom. Syst.* 73 135–143.

[B20] Popov D. Gaponov I. Ryu J.-H. (2016). Portable exoskeleton glove with soft structure for hand assistance in activities of daily living. *IEEE/ASME Trans. Mechatron.* 22 865–875.

[B21] Radder B. Prange-Lasonder G. B. Kottink A. I. Gaasbeek L. Holmberg J. Meyer T. (2016). A wearable soft-robotic glove enables hand support in ADL and rehabilitation: a feasibility study on the assistive functionality. *J. Rehabil. Assist. Technol. Eng.* 3:2055668316670553. 10.1177/2055668316670553 31186913 PMC6453057

[B22] Randazzo L. Iturrate I. Perdikis S. Millán J. D. R. (2017). mano: A wearable hand exoskeleton for activities of daily living and neurorehabilitation. *IEEE Robot. Automat. Lett.* 3 500–507.

[B23] Rodrigue H. Wang W. Kim D.-R. Ahn S.-H. (2017). Curved shape memory alloy-based soft actuators and application to soft gripper. *Comp. Struct.* 176 398–406.

[B24] Susanto E. A. Tong R. K. Ockenfeld C. Ho N. S. (2015). Efficacy of robot-assisted fingers training in chronic stroke survivors: a pilot randomized-controlled trial. *J. Neuroeng. Rehabil.* 12:42. 10.1186/s12984-015-0033-5 25906983 PMC4422529

[B25] Vidal A. F. P. Morales J. Y. R. Torres G. O. Vazquez F. D. S. Rojas A. C. Mendoza J. A. B. (2021). Soft Exoskeletons: Development, Requirements, and Challenges of the Last Decade. *Actuators* 10:166.

[B26] Villoslada A. Flores A. Copaci D. Blanco D. Moreno L. (2015). High-displacement flexible Shape Memory Alloy actuator for soft wearable robots. *Robot. Autonom. Syst.* 73 91–101.

[B27] Wang D. Meng Q. Meng Q. Li X. Yu H. (2018). Design and development of a portable exoskeleton for hand rehabilitation. *IEEE Trans. Neural Syst. Rehabil. Eng.* 26 2376–2386. 30387735 10.1109/TNSRE.2018.2878778

[B28] Wang Y. Zheng S. Pang J. Li S. Li J. (2021). Design and experiment of a hand movement device driven by shape memory alloy wires. *J. Robot.* 2021:6611581.

[B29] Wege A. Hommel G. (2005). “Development and control of a hand exoskeleton for rehabilitation of hand injuries,” in *Proceedings of the 2005 IEEE/RSJ International Conference on Intelligent Robots and Systems*, Piscataway, NJ.

[B30] Xie Q. Meng Q. Zeng Q. Dai Y. Wu Z. Chen L. (2021). Design of a Soft Wrist Exoskeleton Based on SMA Actuator Module. *Jiqiren/Robot* 43 406–413. 10.13973/j.cnki.robot.200584

[B31] Xiloyannis M. Cappello L. Khanh D. B. Yen S.-C. Masia L. (2016). “Modelling and design of a synergy-based actuator for a tendon-driven soft robotic glove,” in *Proceedings of the 2016 6th IEEE International Conference on Biomedical Robotics and Biomechatronics (BioRob)*, Piscataway, NJ.

[B32] Yao Z. Linnenberg C. Argubi-Wollesen A. Weidner R. Wulfsberg J. P. (2017). Biomimetic design of an ultra-compact and light-weight soft muscle glove. *Prod. Eng.* 11 731–743.

[B33] Yap H. K. Lim J. H. Goh J. C. H. Yeow C.-H. (2016). Design of a soft robotic glove for hand rehabilitation of stroke patients with clenched fist deformity using inflatable plastic actuators. *J. Med. Dev.* 10:044504.

[B34] Yap H. K. Lim J. H. Nasrallah F. Yeow C.-H. (2017). Design and preliminary feasibility study of a soft robotic glove for hand function assistance in stroke survivors. *Front. Neurosci.* 11:547. 10.3389/fnins.2017.00547 29062267 PMC5640819

[B35] Yun S.-S. Kang B. B. Cho K.-J. (2017). Exo-glove PM: An easily customizable modularized pneumatic assistive glove. *IEEE Robot. Automat. Lett.* 2 1725–1732.

[B36] Zhang J. (2014). *Bioinspired Design of SMA Artificial Muscle with Actuation, Sensing and Structure.* Shanghai: Shanghai Jiao Tong University.

