# Corrigendum: Design of a SMA-based soft composite structure for wearable rehabilitation gloves

**DOI:** 10.3389/fnbot.2023.1269432

**Published:** 2023-08-08

**Authors:** Qiaolian Xie, Qiaoling Meng, Wenwei Yu, Zhiyu Wu, Rongna Xu, Qingxin Zeng, Zhongchao Zhou, Tianyi Yang, Hongliu Yu

**Affiliations:** ^1^Institute of Rehabilitation Engineering and Technology, University of Shanghai for Science and Technology, Shanghai, China; ^2^Shanghai Engineering Research Center of Assistive Devices, Shanghai, China; ^3^Department of Medical Engineering, Chiba University, Chiba, Japan; ^4^Center for Frontier Medical Engineering, Chiba University, Chiba, Japan

**Keywords:** soft exoskeleton, shape memory alloys (SMA), soft composite structure, hand exoskeleton, SMA actuator

In the published article, there was an error. The finger angles were described as flexed, whereas they should have been depicted as extended.

A correction has been made to 3. Experimental results, *3.4. Experiment of fingers motion angles*, paragraph one. These sentences previously stated:

“The angles of five fingers extension were recorded, as shown in Figure 11B. The motion range of thumb extension is about 100° and its cycle is about 13 s. The motion range of forefinger extension is about 105° and its cycle is about 17 s. The motion range of middle finger extension is about 90° and its cycle is about 15 s. The motion range of extension of the ring and little fingers is about 110° and its cycle is about 19 s.”

The corrected sentences appears below:

“The angles of five fingers extension were recorded, as shown in Figure 11B. The motion range of thumb extension is about 40° and its cycle is about 12 s. The motion range of forefinger extension is about 30° and its cycle is about 13 s. The motion range of middle finger extension is about 40° and its cycle is about 12 s. The motion range of extension of the ring and little fingers is about 40° and its cycle is about 12 s.”

The authors apologize for this error and state that this does not change the scientific conclusions of the article in any way. The original article has been updated.

